# Supplementary material for: Considering hormone-sensitive cancers as a single disease in the UK biobank reveals shared aetiology
Source: Commun Biol. 2022 Jun 21;5:614. doi: 10.1038/s42003-022-03554-y (PMC9213416; doi:10.1038/s42003-022-03554-y)
Supplement: Supplementary file 2 — Supplementary information [file 42003_2022_3554_MOESM2_ESM.pdf]

Supplementary Information

Heritability of Hormone-Sensitive Cancers as a Single Disease in the UK Biobank: A Molecular Evidence of Shared Aetiology

Muktar Ahmed[1,2,3,4]✉, Ville-Petteri Mäkinen[1,5], Anwar Mulugeta[1,3,4], Jisu Shin[1,6], Terry Boyle[1,4,6], Elina Hyppönen[1,3,4,7], Sang Hong Lee[1,4,6,7]✉

[1] Australian Centre for Precision Health, University of South Australia, Adelaide, SA, Australia

[2] Department of Epidemiology, Faculty of Public Health, Jimma University Institute of Health, Jimma Ethiopia

[3] UniSA Clinical and Health Sciences, University of South Australia, Adelaide, SA, Australia

[4] South Australian Health and Medical Research Institute, Adelaide, SA, Australia

[5] Computational Systems Biology Program, Precision Medicine Theme, South Australian Health and Medical Research Institute, Adelaide, SA, Australia

[6] UniSA Allied Health & Human Performance, University of South Australia, Adelaide, SA, Australia

[7] These authors jointly supervised this work: Sang Hong Lee, Elina Hyppönen

Supplementary Table Titles and Summaries

Supplementary Table 1: Meta-analysed SNP-based Heritability estimates using Univariate GREML for subgroups of cancer in UKB [n=288,837]

Supplementary Table 2: SNP-based Heritability estimates for subgroups of cancers in the UKB using LDSC [n=288,837]

Supplementary Table 3: Meta-analysed SNP-based Heritability estimates using univariate GREML for subgroup of incident cancers in UKB [n=288,837]

Supplementary Table 4: SNP-based Heritability estimates for subgroups of incident cancers in the UKB using LDSC [n=288,837]

Supplementary Table 5: Summary of SNP-based heritability estimates using GREML and LDSC for both all cases and Incident only cases in the UKB

Supplementary Table 6: number of cancer cases for each component of hormone-sensitive cancer including incident cases.

Supplementary Table 7: Genome-wide significant SNP for hormone-sensitive cancers in the UKB

Supplementary Table 8: Genome-wide significant SNPs for incident hormone-sensitive cancers in the UKB

Supplementary Table 9: independent loci LD for genome-wide significant SNPs of incident hormone-sensitive cancer GWAS in the UKB

Supplementary Table 10: Genome-wide significant SNPs in meta-analysed single trait hormone-sensitive cancer GWAS in the UK Biobank

Supplementary Table 11: Phenotypic and Genetic correlation for Hormone-sensitive cancers and other non-cancer traits in the UKB using bivariate LDSC

Supplementary Table 12: GxEsum based GxE interaction estimates for incident hormone-sensitive cancers using the baseline measurement characteristics.

Supplementary Fig. 1: Heritability estimates for incident hormone-sensitive cancers in the UK Biobank

Supplementary Fig. 2: LD heatmap for pairwise LD between genome-wide significant SNPs

Supplementary Fig. 3: Quantile-Quantile plot of hormone-sensitive cancers association result in the UKB

Supplementary Fig. 4: Manhattan [left panel] and Quantile-Quantile plot[right panel] for meta-analysed single trait GWAS in the UKB

Supplementary Fig. 5: Schematic diagram of leave-one-out analysis for genetic correlation among hormone-sensitive cancers in the UK Biobank

Supplementary Fig. 6: Power graph for GWAS analysis

Supplementary Note

Heritability estimates:

Heritablity (h^2) is defined as the proportion of traits variation statistically attributed to additive genetic effects. The h^2, is estimated by using a linear mixed-effect model. Usually, such model decomposes the phenotypic variance into genetic and residual variance components. It can be estimated using genetic relationship matrix from common single-nucleotide polymorphisms (SNPs) for unrelated individuals. For qualitative traits i.e., for disease traits, the analysis is usually conducted using an underlying continuous liability wherein the assumption of individuals are to be affected if they exceed a certain liability threshold. The heritability estimate then refers to the heritability on liability scale, rather than the heritability of the observed trait value (1). We have applied two methods in estimating the univariate heritability of grouped level of cancers. First, we applied the univariate GREML analysis (2) using individual-level genotype data in the UKB (UK Biobank). Then, the summary statistics-based estimates in univariate ldsc is applied (3). In both the applied methods the greml estimates of SNP-based heritability was higher as compared to the estimate from ldsc. In the present study we showed that the phenotypic variance for cancer traits were also captured by common SNPs. Among these, we quantified the genetic correlation between traits explained by the common SNPs. This provides an estimate of the heritability explained by common variants because of presumed lesser linkage disequilibrium between the common SNPs and the rest of the genome as compared to related individuals. It is worth noting that ldsc estimate used summary statistics, often from different datasets usually cohorts or data sources suggesting that all of the heritability estimates are subjected to substantial measurement error. Furthermore, LD structure is population-specific, and that LD scores are not stable across the population thereby the LD score are different across the population. In addition, ldsc assumes that heritability is spread across the entire genome making such an approach works best for highly polygenic traits.

GREML-Univariate to estimate heritability:

Genomic-relatedness-matrix restricted maximum likelihood (GREML) refers to the statistical method that estimates the amount of variance in one or more phenotypes that is attributable to a collection of observed genetic polymorphisms. The method is so named because it models phenotypic similarity among individuals in terms of one or more genomic-relatedness matrices (GRMs, described below), and estimates such models via restricted maximum likelihood. GREML

was first implemented in the software GCTA (4), and studies using it have shown that common SNPs can account for a substantial proportion of variance in complex human traits such as height.

In estimating the genetic correlation (r_g) using bivariate LDSC, we use the pre-computed LD score for white Europeans. The GWAS summary statistics are reformatted using the script *munge_sumstats.py* to work with *ldsc*. As we use an imputed quality control (QC) dataset of the UKB, we did not filter the summary statistics to HapMap3 SNPs using - - *merge-alleles* flag in *ldsc*. The sample size for each trait is assigned using - -*N* flag in the script. In each procedure, we checked for any warning message in each log file. To estimate the genetic correlation between the two hormone-sensitive cancer types [Thyroid and Ovarian cancers] with other types of cancers, the summary statistics for the two caners had a mean chi-square value below 1.02 from the *.munge_sumstats.py* warning message due to the smaller sample size. This suggests the data were not suitable for LD score regression and we did not include the genetic correlation from the bivariate LDSC estimate for these two cancer traits. The results for bivariate LDSC genetic correlation are shown in the Supplementary Table 11.

Post-GWAS analysis:

After association analysis to examine the overall genome-wide distribution of test statistics, we constructed the quantile-quantile (QQ) plot for hormone-sensitive cancers in each case [all hormone-sensitive cancers vs incident hormone-sensitive cancer cases only] and quantified the degree of genomic inflation factor (lambda=λ) i.e., how best the observed data points fit to expected, in the post GWAS analysis. The QQ plots in each case showed the bulk of the distribution is in the lower tail of the graph. For all hormone-sensitive cancer cases, we observed a low genomic inflation factor (λ = 1.068365) and similarly for incident hormone-sensitive cancers low genomic inflation factor (λ =1.043601). This further suggests the data follows the normal chi-squared distribution (no inflation) with little population stratification. Usually, λ scales with the sample size, we rescaled the lambda estimate for an equivalent sample size of 1000 cases and controls for better information on the inflation/deflation of the value (See main method). Finally, the rescaled λ for hormone-sensitive cancers is 1.0025325, and for incidence hormone cancers the rescaled λ is 1.00337594, which is closer to 1 in both cases.

The genomic inflation factor is calculated by getting the median p-value first, convert the p-value into chi-squared statistics and finally we calculated the inflation factor using the equation of the following.

$$\lambda GC = \frac{\chi^2\, median}{0.456} \qquad (1)$$

We corss checked the value from the plink output of genomic inflation factor using adjust command during GWAS analysis. The manual computation of the genomic inflation factor is in R using the following script.

Linkage disequilibrium (LD) is a population-based parameter that describes the degree to which an allele of one genetic variants is inherited or correlated with an allele of a nearby genetic variant within a given population.

Covariates

In this study, we used basic sociodemographic, lifestyle-behaviour, and disease information from the UKB baseline assessment. The covariates were age, sex, BMI level, educational status, alcohol consumption, smoking history. All were self reported at baseline, with the exception of BMI, which is derived from body weight and height measurement calculated as body weight [in kilograms] divided by height squared [in meters]. Socioeconomic status was assessed using the Townsend deprivation index. Each participant was assigned a score corresponding to socioeconomic data attributed to their postcode, this was derived from the preceding UK census of population and housing.

Phenotype and Genetic correlation

As cancer phenotype may be modulated by genetic and non-genetic modifiers, we first determine the phenotype correlation of hormone-sensitive cancers with other non-cancer traits. The genetic correlation is a quantitative genetic parameter that describes the genetic relationship between two traits and has been expected to reflect pleiotropic action of genes or correlation between causal loci in two traits. Studies of genetically correlated traits improve our understanding of complex traits including cancer because they can reveal genetic variation that contributes to complex disease, improve genetic prediction and inform therapeutic interventions.

In a general quantitative genetic model, in which, for each individual, two traits (x and y) are each defined as the sum of a genetic value (g) and a residual value (e, with residual simply meaning the difference between the trait value and the genetic value).

$$x = g_x + e_x \qquad (2)$$

$$y = g_y + e_y \qquad (3)$$

The genetic correlation (r_g) of the traits is defined as :

$$\rho_g = \frac{\sigma g_x, g_y}{\sqrt{\sigma_{g_x}^2 \sigma_{g_y}^2}} \qquad (4)$$

Where $\sigma g_x, g_y$ is the covariance of the genetic values and $\sigma^2_{g_x}\sigma^2_{g_y}$ is genetic variance of the two traits in the population. As a result, ρ_g ranges from –1 to 1. Following convention, the Greek letters emphasize parameters (ρ_g), which are replaced by Roman letters for estimates (r_g).

Estimation of the genetic correlation from individual level genotype data involves a bivariate extension of the univariate GREML that uses a genomic relationship matrix (GRM) to estimate SNP-based heritability. As for traditional epidemiology data, this approach uses a Linear Mixed Model (LMM), in which the phenotype is modelled as a function of the genetic values of individuals, but the variance–covariance structure of genetic values is described by genetic relationships in the GRM constructed from observed genome-wide SNP data rather than from pedigree data. SNP-based heritability is expected to be lower than heritability estimated from epidemiological family records because it aims to capture only causal variation that is in linkage disequilibrium (LD) with the measured SNPs.

In the analysis between hormone-sensitive cancers and non-cancer traits using GWAS summary statistics a nominally significant correlation has been found. For example, IGF-1 positively correlated with the development of hormone-sensitive cancer in all combined cases [incident and prevalent cases of hormone-sensitive cancer]. This suggests IGF-1 is a common factor that directly affects hormone-sensitive cancer risk as well as increasing height. Furthermore, a slightly significant negative genetic correlation with SHBG (rg = -0.1141, se=0.0465; (P=1.42E-02)) was observed. Even though the estimates were non-significant for the rest of the non-cancer traits, there was slightly higher and a meaningful direction of estimates in comparison to previous observational studies. For example, a slightly higher positive genetic correlation was found between hormone-sensitive cancers and menopausal status (rg =0.1183, se=0.1027; (P=2.49E-01)); T2D status (rg =0.0839, se=0.0628; (P=1.82E-01)); baseline LDL cholesterol measurement (rg = 0.0695, se=0.0635; (P=2.74E-01)); WHR adjusted for BMI (rg =0.0675, se=0.0446; (P=1.30E-01)); alcohol consumption (rg =0.0531, se=0.0857; (P=5.36E-01)); and apolipoprotein B (rg =0.0638, se=0.0596; (P=2.84E-01)). In contrast, evidence of higher negative genetic correlation has also been observed between hormone-sensitive cancers and in non-cancer traits such as diastolic blood pressure (rg = -0.0512, se=0.0487; (P=2.93E-01)); BMI (rg = -0.0504, se=0.0417; (P=2.27E-01)); testosterone (rg = -0.0461, se=0.0477; (P=3.36E-01)) and apolipoprotein A1 (rg = -0.0390, se=0.0428; (P=3.62E-01)). The genetic correlations estimate between hormone-sensitive cancer and other non-cancer traits is presented in Supplementary Table 11.

Gene environment interaction (GxE)

GxE tests can explain novel biology along two distinct, complementary axes. First, GxE can identify unappreciated genetic effect that elude linear models, which can increase GWAS power and has recently received attention as a partial answer to the missing heritability question. Second, GxE test can demonstrate that an environmental measurement is biologically trait relevant and quantify its impact, which can be important for public health and can illuminate intrinsic traits biology (Supplementary Table 12).

Supplementary Tables

The following supplementary results are from the detailed analysis we tested in each section of the main text. They are presented here for further references.

Supplementary Table 1: Meta-analysed SNP-based Heritability estimates using Univariate GREML for subgroups of cancer in UKB [n=288,837]

Univariate GREML SNP-h^2 of cancer [incident and prevalent cases]in the UK Biobank UKB-1				
	Liability scale			
Cancer Types	h^2	SE	χ^2 test	Sig.
Overall Cancer	0.0438	0.0031	194.524	3.27E-44
Non-obesity cancer	0.0169	0.0048	12.246	4.66E-04
Obesity-related	0.0527	0.0048	120.648	4.56E-28
Hormonal Cancer	0.1006	0.0070	204.565	2.11E-46
Non-hormonal Cancer	0.0307	0.0307	18.048	2.15E-05

The observed heritability is transformed into the liability scale in MTG2.17 using the prevalence (k) of cases in each subgroup of cancer. The model is adjusted for batch effect, assessment centre, the 10 ancestry-informative principal components (PCs), birthplace, age, sex, smoking, alcohol, education & TDI

Supplementary Table 2: SNP-based Heritability estimates for subgroups of cancers in the UKB using LDSC [n=288,837]

Univariate LDSC SNP-h^2 of cancer [incident and prevalent cases]in the UK Biobank				
	Liability scale			
Cancer Types	h^2	SE	χ^2 test	Sig.
Overall Cancer	0.0330	0.0046	51.46503	7.29E-13
Non-obesity cancer	0.0149	0.0113	1.738664	1.87E-01
Obesity-related	0.0413	0.0065	40.37136	2.10E-10
Hormonal Cancer	0.0699	0.0083	70.92481	3.71E-17
Non-hormonal Cancer	0.0300	0.0101	8.822664	2.98E-03

The observed heritability is transformed into the liability scale in MTG2.17 using the prevalence (k) of cases in each subgroup of cancer. The model is adjusted for batch effect, assessment centre, 10 ancestry-informative principal components (PCs), birthplace, age, sex, smoking, alcohol, education & TDI

Supplementary Table 3: Meta-analysed SNP-based Heritability estimates using univariate GREML for subgroup of incident cancers in UKB [n=288,837]

Univariate GREML SNP-h2 of incident cancers in the UK Biobank (Liability Scale) UKB				
Cancer Types	h^2	SE	χ^2 test	Sig.
Overall Cancer	0.0438	0.0031	194.5240	3.27E-44
Non-obesity cancer	0.0169	0.0048	12.2462	4.66E-04
Obesity-related	0.0526	0.0047	120.6483	4.56E-28
Hormonal Cancer	0.1006	0.0070	204.5651	2.11E-46
Non-hormonal Cancer	0.0306	0.0072	18.0485	2.15E-05

Adjustment is made for batch effect, assessment centre, 10 ancestry-informative principal components (PCs), birthplace, age, sex, smoking, alcohol, education, & TDI.

Supplementary Table 4: SNP-based Heritability estimates for subgroups of incident cancers in the UKB using LDSC [n=288,837]

Univariate GREML SNP-h2 of incident cancers in the UK Biobank (Liability Scale) UKB-1				
Cancer Types	h^2	SE	χ^2 test	Sig.
Overall Cancer	0.0184	0.0072	6.530864	1.06E-02
Non-obesity cancer	0.0097	0.0250	0.150544	6.98E-01
Obesity-related	0.0169	0.0089	3.605732	5.76E-02
Hormonal Cancer	0.0560	0.0158	12.56209	3.94E-04
Non-hormonal Cancer	0.0066	0.0158	0.174491	6.76E-01

The observed heritability is transformed into the liability scale in MTG2.17 using the prevalence (k) of cases in each subgroup of cancer. Adjustment is made for batch effect, assessment centre, 10 ancestry-informative principal components (PCs), birthplace, age, sex, smoking, alcohol, education, & TDI.

Supplementary Table 5: Summary of the SNP-based heritability estimates using GREML and LDSC for both all cases and Incident only cases in the UKB

	Prevalent and incident cases								Incident cases only							
**Method**	**Observed scale**				**Liability scale**				**Observed scale**				**Liability scale**			
**GREML(UKB)**	**h^2**	**SE**	**Wald**	**p-value**	**h^2**	**SE**	**Wald**	**P-value**	**h^2**	**SE**	**Wald**	**p-value**	**h^2**	**SE**	**Wald**	**p-value**
Overall-Cancer	0.0180	0.0013	193.1707	6.46E-44	0.0438	0.0030	194.5240	3.27E-44	0.0088	0.0014	41.8902	9.65E-11	0.0313	0.0048	41.9656	9.29E-11
Obesity-related	0.0148	0.0014	119.3296	8.87E-28	0.0526	0.0047	120.648	4.56E-28	0.0066	0.0014	22.4836	2.12E-06	0.0343	0.0072	22.5215	2.08E-06
Non-Obesity	0.0049	0.0014	12.2655	4.61E-04	0.0169	0.0048	12.2462	4.66E-04	0.0008	0.0014	0.3368	5.62E-01	0.0043	0.0075	0.32828	5.67E-01
Hormonal	0.0204	0.0014	203.7643	3.15E-46	0.1006	0.0070	204.565	2.11E-46	0.0077	0.0014	28.8187	7.95E-08	0.0592	0.0110	28.84536	7.84E-08
Non-Hormonal	0.0059	0.0014	18.0814	2.12E-05	0.0306	0.0072	18.0485	2.15E-05	0.0032	0.0014	4.9695	2.58E-02	0.0326	0.0146	4.97122	2.58E-02
**LDSC**	**h^2**	**SE**	**Wald**	**p-value**	**h^2**	**SE**	**Wald**	**p-value**	**h^2**	**SE**	**Wald**	**p-value**	**h^2**	**SE**	**Wald**	**p-value**
Overall	0.0136	0.0019	51.23546	8.19E-13	0.0330	0.0046	51.46503	7.29E-13	0.0051	0.0020	6.5025	1.08E-02	0.0184	0.0072	6.5308	1.06E-02
Obesity-related	0.0133	0.0021	40.1111	2.40E-10	0.0413	0.0065	40.37136	2.10E-10	0.0038	0.0020	3.61	5.74E-02	0.0169	0.0089	3.6057	5.76E-02
Non-Obesity	0.0029	0.0022	1.737603	1.87E-01	0.0149	0.0113	1.738664	1.87E-01	0.0007	0.0018	0.15123	6.97E-01	0.0097	0.0250	0.1505	6.98E-01
Hormonal	0.0176	0.0021	70.24036	5.25E-17	0.0699	0.0083	70.92481	3.71E-17	0.0067	0.0019	12.4349	4.21E-04	0.0560	0.0158	12.562	3.94E-04
Non-Hormonal	0.0062	0.0021	8.716553	3.15E-03	0.0300	0.0101	8.822664	2.98E-03	0.0008	0.0019	0.1772	6.74E-01	0.0066	0.0158	0.1744	6.76E-01

Supplementary Table 6: number of cancer cases for each component of hormone-sensitive cancer including incident cases in the UKB.

No	Cancer types	All cases	Incident cases
1	Post menopausal breast cancer	7,090	2,326
2	Prostate cancer	5,643	3,665
3	Ovarian cancer	1,063	422
4	Uterine cancer	1,036	501
5	Thyroid cancer	365	124
	**Total cases**	**15,197**	**7,038**

The number of each cancer cases in the hormone-sensitive cancer incident and all cases (incident and prevalent cases).

Supplementary Table 7: Genome-wide significant SNP for hormone-sensitive cancers in the UKB

No	CHR	SNP	BP	A1	NMISS	BETA	STAT	P	Gene name	Genomic region	consequence	Trait Association
1	2	rs13387042	217905832	G	237570	-0.003843	-5.64	1.70E-08	AC007749.1	2q35		Breast Cancer
2	8	rs10086908	128011937	C	236744	-0.004774	-6.406	1.50E-10	PCAT1	8q24.21		Prostate Cancer
3	8	rs16901898	128015092	C	236819	-0.00475	-6.377	1.81E-10				None
4	8	rs9297750	128022973	G	236791	-0.00512	-6.494	8.35E-11				None
5	8	rs17762878	128025053	G	237572	-0.004702	-6.312	2.76E-10				None
6	8	rs7823764	128026262	G	237501	-0.004753	-6.38	1.78E-10				None
7	8	rs7842175	128026317	C	237507	-0.00474	-6.362	1.99E-10				None
8	8	rs1016343	128093297	T	237567	0.004683	5.536	3.09E-08	PRNCR1	8q24.21		Prostate Cancer
9	8	rs16901949	128107153	C	237553	0.01063	5.616	1.96E-08				None
10	8	rs16901959	128109530	G	237549	0.01057	5.586	2.33E-08				None
11	8	rs7830341	128109930	A	237497	0.01058	5.585	2.34E-08	CASC19	8q24.21		BMI
12	8	rs16901966	128110252	G	237519	0.01061	5.604	2.09E-08				None
13	8	rs16901970	128112715	G	237549	0.01057	5.586	2.33E-08				None
14	8	rs7824785	128114710	T	237464	0.01052	5.565	2.62E-08				None
15	8	rs16901979	128124916	A	237573	0.01046	5.536	3.10E-08	CASC19	8q24.21		Prostate Cancer
16	8	rs10505483	128125195	T	237573	0.01048	5.55	2.87E-08	CASC19	8q24.21		Prostate Cancer
17	8	rs7817677	128125504	G	237543	0.01056	5.581	2.40E-08				None
18	8	rs6989838	128129372	C	237551	0.01053	5.574	2.49E-08				None
19	8	rs10505477	128407443	G	237573	-0.003757	-5.508	3.63E-08	POU5F1B	8q24.21		Colorectal & Prostate
20	8	rs6983267	128413305	T	237573	-0.003998	-5.859	4.67E-09	POU5F1B	8q24.21		Colorectal & Prostate
21	8	rs4242382	128517573	A	237573	0.006448	5.722	1.06E-08	AC104370.1	8q24.21		Prostate Cancer
22	8	rs4242384	128518554	C	237573	0.00649	5.746	9.16E-09	AC104370.1	8q24.21		Prostate Cancer
23	8	rs7814837	128522202	T	237341	0.006404	5.66	1.52E-08				None
24	8	rs7824868	128524414	T	235426	0.006156	5.489	4.06E-08				None
25	10	rs10993994	51549496	T	237568	0.004194	6.01	1.86E-09	MSMB	10q11.22		Blood protein & Prostate
26	10	rs10736303	123334457	G	236037	0.004702	6.856	7.08E-12	FGFR2			None
27	10	rs11200014	123334930	A	236681	0.005077	7.295	2.99E-13	FGFR2	10q26.13		Prostate Cancer

28	10	rs2981579	123337335	A	237572	0.005131	7.388	1.50E-13	FGFR2	10q26.13		Breast Cancer
29	10	rs1078806	123338975	G	236200	0.005161	7.407	1.30E-13	FGFR2	10q26.13		ECG & Breast cancer
30	10	rs2981575	123346116	G	237572	0.005487	7.863	3.76E-15	FGFR2	10q26.13		Breast & Prostate cancer
31	10	rs1219648	123346190	G	237572	0.005456	7.815	5.51E-15	FGFR2	10q26.13		Breast Cancer
32	10	rs2912774	123348662	T	236629	0.005355	7.66	1.87E-14	FGFR2	10q26.13		Breast Cancer
33	10	rs2420946	123351324	T	236506	0.005036	7.174	7.31E-13	FGFR2	10q26.13		Breast Cancer
34	10	rs2162540	123352136	C	237281	0.004956	7.068	1.58E-12	FGFR2			None
35	10	rs2981582	123352317	A	237573	0.004969	7.088	1.37E-12	FGFR2	10q26.13		Breast Cancer
36	11	rs4255548	68973970	A	231722	-0.004173	-5.887	3.93E-09			Intergenic variant	None
37	11	rs7929962	68985583	C	236975	-0.004097	-6.009	1.87E-09	AP003071.2	11q13.3		Prostate Cancer
38	11	rs7109672	68991110	A	237475	-0.003999	-5.867	4.45E-09				None
39	11	rs7931342	68994497	T	237573	-0.004042	-5.932	3.00E-09	AP003071.2	11q13.3		Prostate Cancer
40	11	rs10896449	68994667	A	237566	-0.004077	-5.974	2.32E-09	AP003071.2	11q13.3		Prostate Cancer
41	11	rs7130881	68995958	G	237570	0.005563	6.15	7.75E-10	AP003071.5	11q13.3		Prostate Cancer
42	11	rs9787877	68996509	T	237336	-0.004082	-5.989	2.11E-09				None
43	11	rs11603288	68996782	A	227932	-0.004617	-5.512	3.54E-08				None
44	11	rs7939250	69002950	G	235313	-0.004053	-5.92	3.23E-09				None
45	11	rs10896450	69008114	A	234368	-0.004109	-5.989	2.12E-09				None
46	11	rs12799883	69010651	T	232146	-0.004056	-5.881	4.08E-09				None
47	16	rs17271951	52538040	C	234844	0.005202	6.566	5.19E-11				None
48	16	rs4784223	52575907	G	236768	0.005134	6.56	5.41E-11	TOX3	16q12.1		Breast Cancer
49	16	rs3803662	52586341	A	237573	0.004882	6.261	3.83E-10	CASC16	16q12.1		Breast Cancer
50	16	rs4784227	52599188	T	237573	0.005807	7.267	3.70E-13	CASC16	16q12.2		Breast & Parkinson's
51	16	rs12922061	52635000	T	235775	0.005089	6.198	5.73E-10	CASC16	16q12.2		Breast Cancer
52	17	rs4430796	36098040	G	237530	-0.003838	-5.611	2.01E-08	HNF1B	17q12		Endometrial, Prostate & T2D
53	17	rs11651755	36099840	C	233456	-0.003835	-5.571	2.54E-08	HNF1B	17q12		Ovarian & T2D
54	17	rs11263763	36103565	G	232849	-0.003894	-5.642	1.68E-08	HNF1B	17q12		Endometrial and Prostate
55	19	rs17632542	51361757	C	237572	-0.00831	-6.359	2.03E-10	KLK3	19q13.33		Prostate Cancer

None means the identified SNP is not found in GWAS catalogue

Supplementary Table 8: Genome-wide significant SNPs for incident hormone-sensitive cancers in the UKB

No	CHR	SNP	BP	A1	TEST	NMISS	BETA	STAT	P	Trait Association	Cross-Traits		Other Population
1	8	rs10086908	128011937	C	ADD	229054	-0.003004	-5.597	2.19E-08	Prostate			
2	8	rs16901898	128015092	C	ADD	229127	-0.003008	-5.609	2.04E-08	None			
3	8	rs9297750	128022973	G	ADD	229094	-0.003338	-5.882	4.05E-09	None			
4	8	rs17762878	128025053	G	ADD	229856	-0.002971	-5.539	3.05E-08	None			
5	8	rs7823764	128026262	G	ADD	229788	-0.003011	-5.612	2.01E-08	None			
6	8	rs7842175	128026317	C	ADD	229793	-0.003011	-5.612	2.00E-08	None			
7	8	rs1016343	128093297	T	ADD	229851	0.003468	5.69	1.27E-08	Prostate			
8	8	rs16901949	128107153	C	ADD	229838	0.007865	5.762	8.31E-09	None			
9	8	rs16901959	128109530	G	ADD	229835	0.007862	5.76	8.40E-09	None			
10	8	rs7830341	128109930	A	ADD	229787	0.007959	5.824	5.75E-09	Prostate			
11	8	rs16901966	128110252	G	ADD	229805	0.007884	5.774	7.74E-09	None			
12	8	rs16901970	128112715	G	ADD	229835	0.007862	5.76	8.40E-09	None			
13	8	rs7824785	128114710	T	ADD	229751	0.007881	5.78	7.47E-09	None			
14	8	rs16901979	128124916	A	ADD	229857	0.007712	5.657	1.54E-08	Prostate			
15	8	rs10505483	128125195	T	ADD	229857	0.007815	5.739	9.53E-09	Prostate			
16	8	rs7817677	128125504	G	ADD	229828	0.007923	5.806	6.41E-09	None			
17	8	rs6989838	128129372	C	ADD	229835	0.007818	5.738	9.61E-09	None			
18	8	rs10505477	128407443	G	ADD	229857	-0.002831	-5.762	8.31E-09	Prostate			
19	8	rs6983267	128413305	T	ADD	229857	-0.002841	-5.78	7.50E-09	Prostate	Colon		
20	8	rs7014346	128424792	A	ADD	229857	0.002789	5.495	3.92E-08	Prostate			
21	8	rs1447295	128485038	A	ADD	229856	0.004518	5.565	2.63E-08	Prostate			
22	8	rs1447296	128495359	T	ADD	228589	0.004558	5.605	2.09E-08	None			Prostate and colon
23	8	rs4242382	128517573	A	ADD	229857	0.004903	6.036	1.58E-09	Prostate			
24	8	rs4242384	128518554	C	ADD	229857	0.00495	6.081	1.20E-09	Prostate			
25	8	rs7814837	128522202	T	ADD	229628	0.004893	6.001	1.96E-09	None			Prostate and colon
26	8	rs7824868	128524414	T	ADD	227776	0.004772	5.903	3.58E-09	None			Prostate and colon
27	8	rs9656816	128534654	G	ADD	226576	0.004829	5.457	4.86E-08	None			Prostate and colon
28	8	rs7837688	128539360	T	ADD	229857	0.004446	5.489	4.04E-08	Prostate			

29	10	rs10993994	51549496	T	ADD	229852	0.002802	5.574	2.50E-08	Prostate	MSMB		
30	11	rs7130881	68995958	G	ADD	229854	0.003862	5.923	3.16E-09	Prostate			
31	17	rs4430796	36098040	G	ADD	229818	-0.00291	-5.907	3.49E-09	Prostate	T2DM	Endometrial	
32	17	rs11651755	36099840	C	ADD	225879	-0.002834	-5.712	1.12E-08	Ovarian	T2DM		
33	17	rs11263763	36103565	G	ADD	225280	-0.002853	-5.737	9.67E-09	Prostate	Endometrial		

None means the identified SNP is not found in GWAS catalogue

Supplementary Table 9: independent loci LD for genome-wide significant SNPs of incident hormone-sensitive cancer GWAS in the UKB

No	CHR	BP	SNP	A1	P	BETA	STAT	NMISS
1	8	128011937	rs10086908	C	2.19E-08	-0.003004	-5.597	229,054
2	8	128093297	rs1016343	T	1.27E-08	0.003468	5.690	229,851
3	8	128107153	rs16901949	C	8.315-09	0.007865	5.762	229,838
4	8	128407443	rs10505477	G	9.53E-09	0.007815	5.739	229,857
5	8	128485038	rs1447295	A	2.63E-08	0.004518	5.565	229,856
6	10	51549496	rs10993994	T	2.50E-08	0.002802	5.574	229,852
7	11	68995958	rs7130881	G	3.16E-09	0.003862	5.923	229,854
8	17	36098040	rs4430796	G	3.49E-09	-0.002910	-5.907	229,818

Abbreviations: BP**:** Base-pair position; A1=First allele code; P= Fixed-effect meta-analysis P-value; BETA = Fixed-effects Beta estimate

Supplementary Table 10: Genome-wide significant SNPs in meta-analysed single trait hormone-sensitive cancer GWAS in the UK Biobank

No	CHR	BP	SNP	A1	N	P	P.R.	BETA	BETA.R.	Q	I
1	9	100534147	rs1877431	A	5	1.70E-12	9.54E-02	8.00E-04	4.00E-04	8.19E-02	51.68
2	9	100535267	rs7030280	C	5	1.36E-18	1.10E-02	1.00E-03	7.00E-04	8.70E-02	50.79
3	9	100537455	rs10983700	T	5	2.88E-18	2.25E-02	1.00E-03	7.00E-04	6.29E-02	55.20
4	9	100537802	rs1588635	A	5	4.48E-18	1.82E-02	1.00E-03	7.00E-04	7.33E-02	53.23
5	9	100546391	rs925488	G	5	3.05E-18	2.41E-02	1.00E-03	6.00E-04	6.09E-02	55.60
6	9	100549013	rs7850258	A	5	2.96E-18	2.32E-02	1.00E-03	6.00E-04	6.23E-02	55.32
7	9	100552559	rs10739496	C	5	4.62E-18	3.18E-02	1.00E-03	6.00E-04	5.17E-02	57.49
8	9	100556109	rs965513	A	5	2.28E-18	2.91E-02	1.00E-03	6.00E-04	4.98E-02	57.88
9	9	100556972	rs10759944	A	5	1.37E-18	1.53E-02	1.00E-03	7.00E-04	6.84E-02	54.15
10	9	100574120	rs7870795	T	5	3.27E-09	3.88E-01	7.00E-04	3.00E-04	3.60E-03	74.36
11	9	100575888	rs7859751	G	5	4.21E-09	4.19E-01	7.00E-04	3.00E-04	2.90E-03	75.10
12	9	100583195	rs1443432	C	5	1.10E-11	2.19E-01	7.00E-04	4.00E-04	2.19E-02	65.09
13	9	100595238	rs7024345	A	5	2.75E-11	4.43E-01	8.00E-04	3.00E-04	8.00E-04	79.04
14	9	100596439	rs7043885	C	5	1.87E-09	4.20E-01	7.00E-04	3.00E-04	2.09E-02	65.40
15	9	100608682	rs12348691	G	5	1.26E-09	4.33E-01	7.00E-04	3.00E-04	1.73E-02	66.67
16	9	100608745	rs10759960	A	5	1.30E-09	4.81E-01	7.00E-04	3.00E-04	7.00E-04	79.39
17	9	100608980	rs13288000	T	5	1.27E-09	4.35E-01	7.00E-04	3.00E-04	1.74E-02	66.67
18	9	100609230	rs7873389	C	5	1.30E-09	4.35E-01	7.00E-04	3.00E-04	1.72E-02	66.73
19	9	100612807	rs1348386	A	5	1.37E-11	4.88E-01	8.00E-04	3.00E-04	5.00E-04	80.18
20	9	100614296	rs4743138	A	5	6.35E-11	4.79E-01	8.00E-04	3.00E-04	8.00E-04	78.89
21	9	100615117	rs907577	C	5	2.92E-09	4.24E-01	6.00E-04	3.00E-04	2.36E-02	64.54
22	9	100615949	rs1867278	C	5	2.85E-09	4.19E-01	6.00E-04	3.00E-04	2.40E-02	64.40
23	9	100617479	rs1443434	G	5	3.02E-09	4.31E-01	6.00E-04	2.00E-04	2.23E-02	64.96
24	9	100617583	rs1443435	T	5	5.97E-09	4.19E-01	6.00E-04	2.00E-04	2.84E-02	63.12
25	9	100622597	rs907580	T	5	2.15E-10	5.13E-01	8.00E-04	3.00E-04	8.00E-04	78.87
26	9	100623377	rs993501	G	5	2.33E-10	4.44E-01	7.00E-04	2.00E-04	1.87E-02	66.19
27	9	100625193	rs10759975	T	5	2.33E-10	4.38E-01	7.00E-04	3.00E-04	1.94E-02	65.92
28	9	100634751	rs1955145	C	5	1.82E-10	3.97E-01	7.00E-04	3.00E-04	2.34E-02	64.60
29	9	100636398	rs925487	C	5	1.75E-10	3.97E-01	7.00E-04	3.00E-04	2.35E-02	64.56
30	9	100639275	rs10984103	A	5	1.78E-10	3.97E-01	7.00E-04	3.00E-04	2.28E-02	64.78
31	9	100650096	rs7866436	G	5	4.88E-11	3.38E-01	7.00E-04	3.00E-04	2.52E-02	64.04
32	9	100652582	rs10512255	G	5	9.00E-11	4.16E-01	7.00E-04	3.00E-04	1.78E-02	66.48
33	9	100654093	rs7034648	C	5	9.48E-11	4.26E-01	7.00E-04	3.00E-04	1.74E-02	66.66
34	9	100657119	rs10115216	T	5	9.37E-11	4.10E-01	7.00E-04	3.00E-04	1.81E-02	66.37
35	9	100663700	rs3824495	C	5	7.34E-11	4.25E-01	7.00E-04	3.00E-04	1.66E-02	66.94
36	9	100666543	rs9299258	T	5	7.32E-11	4.23E-01	7.00E-04	3.00E-04	1.67E-02	66.90
37	9	100667599	rs1561961	C	5	7.73E-11	4.23E-01	7.00E-04	3.00E-04	1.75E-02	66.61

Abbreviations: BP**:** Base-pair position; A1=First allele code; N= Number of valid studies for the specific SNP; P= Fixed-effect meta-analysis P-value; P(R)= Random-effects meta-analysis P-value; BETA = Fixed-effects Beta estimate; BETA.R = Random-effects Beta estimates; Q= P-value for Cochrane's Q-statistic; I=I^2 hetetrogeneity index (ranges 0-100); Weighted_Z = weighted Z score value; P(WZ) = weighted Z-score-based P-value.

Supplementary Table 11: Phenotypic and Genetic correlation for Hormone-sensitive cancers and other non-cancer traits using Bivariate LDSC in the UKB

Traits	All hormone-sensitive cancer cases combined (Incident and Prevalent)						Incident hormonal cancers cases					
	Phenotypic correlation			Genetic correlation			Phenotypic correlation			Genetic correlation		
**Glycaemic Traits**	r_p	SE	*p*-value	r_g	SE	*p*-value	r_p	SE	*p*-value	r_g	SE	*p*-value
T2D	0.0084	0.0020	4.49E-05*	0.0839	0.0628	1.82E-01	0.0072	0.0021	9.33E-04*	0.0830	0.1041	4.25E-01
Glucose	0.0022	0.0022	3.13E-01	0.0131	0.0641	8.38E-01	-0.0050	0.0022	2.85E-02*	0.0414	0.0973	6.70E-01
HbA1c	-0.0015	0.0021	4.50E-01	0.0143	0.0473	7.62E-01	-0.0075	0.0021	4.89E-04*	0.0576	0.0779	4.60E-01
**Anthropometric Traits**	r_p	SE	*p*-value	r_g	SE	*p*-value	r_p	SE	*p*-value	r_g	SE	*p*-value
BMI	0.0059	0.0020	4.05E-03*	-0.0504	0.0417	2.27E-01	0.0054	0.0020	8.91E-03*	-0.0979	0.0663	1.40E-01
WHR	0.0064	0.0020	1.78E-03*	0.0196	0.0419	6.40E-01	0.0033	0.0021	1.14E-01	0.0044	0.0636	9.45E-01
WHRadjBMI	0.0053	0.0020	9.68E-03*	0.0675	0.0446	1.30E-01	0.0028	0.0021	1.70E-01	0.0979	0.063	1.20E-01
WC	0.0106	0.0020	2.36E-07*	0.0040	0.0418	9.24E-01	0.0068	0.0020	1.01E-03*	-0.0435	0.0642	4.98E-01
Height (standing)	0.0130	0.0020	1.78E-10*	0.0582	0.0351	9.73E-02*	0.0089	0.0021	2.11E-05*	0.0058	0.0516	9.11E-01
Body fat percentage	0.0058	0.0021	5.04E-03*	-0.0286	0.0411	4.87E-01	0.0045	0.0021	3.41E-02*	-0.0666	0.0661	3.14E-01
**Lipid Profile**	r_p	SE	*p*-value	r_g	SE	*p*-value	r_p	SE	*p*-value	r_g	SE	*p*-value
Cholesterol	0.0045	0.0021	3.28E-02*	0.0445	0.0566	4.32E-01	-0.0037	0.0021	8.26E-02	0.0873	0.091	3.37E-01
Triglyceride	0.0067	0.0021	1.45E-03*	-0.0270	0.0491	5.82E-01	-0.0059	0.0021	5.23E-03*	-0.0124	0.0687	8.57E-01
HDL	-0.0051	0.0022	2.10E-02*	-0.0058	0.0421	8.90E-01	-0.0054	0.0022	2.68E-02*	-0.0678	0.0639	2.89E-01
LDL	0.0051	0.0021	1.39E-02*	0.0695	0.0635	2.74E-01	-0.0014	0.0021	5.30E-01	0.1571	0.1080	1.46E-01
**Behavioural-Lifestyle**	r_p	SE	*p*-value	r_g	SE	*p*-value	r_p	SE	*p*-value	r_g	SE	*p*-value
Alcohol	0.0004	0.0080	8.22E-01	0.0531	0.0857	5.36E-01	-2.48E-11	0.0021	1.00	0.0341	0.1267	7.88E-01
Smoking	-0.0001	0.0020	9.32E-01	-0.0328	0.0479	4.93E-01	2.24E-11	0.0021	1.00	-0.0238	0.0778	7.60E-01
Education	0.0013	0.0020	5.16E-01	0.0339	0.0394	3.90E-01	6.80E-11	0.0021	1.00	0.1271	0.0672	5.86E-02*
Townsend	-1.28E-05	0.0020	9.95E-01	0.0219	0.0906	8.09E-01	-1.00E-11	0.0020	1.00	-0.1341	0.1422	3.46E-01
**Cardiac Traits**	r_p	SE	*p*-value	r_g	SE	*p*-value	r_p	SE	*p*-value	r_g	SE	*p*-value
Systolic Blood Pressure	0.0018	0.0021	4.00E-01	-0.0365	0.0439	4.06E-01	-0.0015	0.0021	4.93E-01	-0.0784	0.068	2.49E-01
Diastolic Blood Pressure	0.0067	0.0021	1.48E-03*	-0.0512	0.0487	2.93E-01	0.0023	0.0021	2.74E-01	-0.1209	0.0722	9.40E-02*
Cardiovascular Disease	-0.0090	0.0020	9.64E-06*	0.0242	0.0703	7.31E-01	-0.0061	0.0021	3.35E-03*	0.0572	0.1134	6.14E-01

C-reactive Protein	0.0080	0.0021	1.46E-04*	-0.0217	0.0580	7.08E-01	-0.0005	0.0021	8.16E-01	-0.0977	0.0877	2.65E-01
Vitamin D	0.0048	0.0021	2.40E-02*	-0.0181	0.0458	6.93E-01	0.0048	0.0021	2.68E-02*	0.0451	0.0791	5.69E-01
**Menstrual Factors**	**r_p**	**SE**	***p*-value**	**r_g**	**SE**	***p*-value**	**r_p**	**SE**	***p*-value**	**r_g**	**SE**	***p*-value**
Menopausal Status	0.0009	0.0035	7.97E-01	0.1183	0.1027	2.49E-01	0.0077	0.0036	3.48E-02*	0.1748	0.1600	2.75E-01
**Cancer-related**	**r_p**	**SE**	***p*-value**	**r_g**	**SE**	***p*-value**	**r_p**	**SE**	***p*-value**	**r_g**	**SE**	***p*-value**
SHBG	-0.0059	0.0022	7.35E-03*	-0.1141	0.0465	1.42E-02*	-0.0086	0.0022	1.20E-04*	-0.0994	0.0776	2.00E-01
Testosterone	-0.0050	0.0022	2.13E-02*	-0.0461	0.0477	3.36E-01	0.0068	0.0022	2.15E-03*	0.0587	0.0800	4.62E-01
Oestradiol	-0.0190	0.0022	2.20E-16*	0.079	0.1476	5.92E-01	0.0025	0.0022	2.68E-01	0.1605	0.2328	4.90E-01
IGF-1	0.0094	0.0021	7.30E-06*	0.0367	0.042	3.82E-01	0.0102	0.0021	2.00E-06*	-0.0044	0.0645	9.45E-01
**Other**	**r_p**	**SE**	***p*-value**	**r_g**	**SE**	***p*-value**	**r_p**	**SE**	***p*-value**	**r_g**	**SE**	***p*-value**
APOA1	-0.0005	0.0022	8.21E-01	-0.0390	0.0428	3.62E-01	-0.0065	0.0022	3.83E-03*	-0.1182	0.0691	8.72E-02*
APOB	0.0050	0.0021	1.61E-02*	0.0638	0.0596	2.84E-01	-0.0017	0.0021	4.08E-01	0.1578	0.0989	1.11E-01

An asterisk indicates significance with P<0.05 using two tailed hypothesis test and normal distribution of the Fischer transformed correlation coefficient. The estimates are reported with their respective standard error. Abbreviations: r_p: phenotypic correlation; r_g: genotypic correlation; SE: standard error; T2D: type II diabetes; HbA1c: glycate haemoglobin; BMI: body mass index; WHR: waist to hip ratio; WC: waist circumference; HDL: high density lipoprotein; LDL: low density lipoprotein; ApoA1: apolipoprotein A 1; ApoB: apolipoprotein B; SHBG: Sex hormone binding globulin.

Supplementary Table 12: GxEsum based GxE interaction estimates for incident hormone-sensitive cancers using the baseline measurement characteristics.

Environment	h^2(se)	GxE Interaction variance (se)	χ^2 test	*P*-value
Waist circumference	5.14% (1.43%)	0.25% (0.18%)	1.92901	1.65E-01
Townsend deprivation index	4.99% (1.43%)	-0.15% (0.18%)	0.69444	4.05E-01
Apolipoprotein B	4.92% (1.56%)	-0.31% (0.19%)	2.66205	1.03E-01
IGF-1	4.30% (1.56%)	-0.03% (0.18%)	0.02777	8.68E-01
Physical activity	6.07% (1.97%)	-0.41% (0.21%)	3.81179	5.09E-02

Abbreviations: IGF-1: insulin-like growth factor; WC: waist circumference. The traits used as environment to detect the interaction are baseline measured waist circumference, Townsend deprivation index, apolipoprotein B, IGF-1, physical activity. Cases included are incident hormone-sensitive cancer cases. Heritability is estimated from the additive model in GxEsum using univariate ldsc.

Supplementary Figures

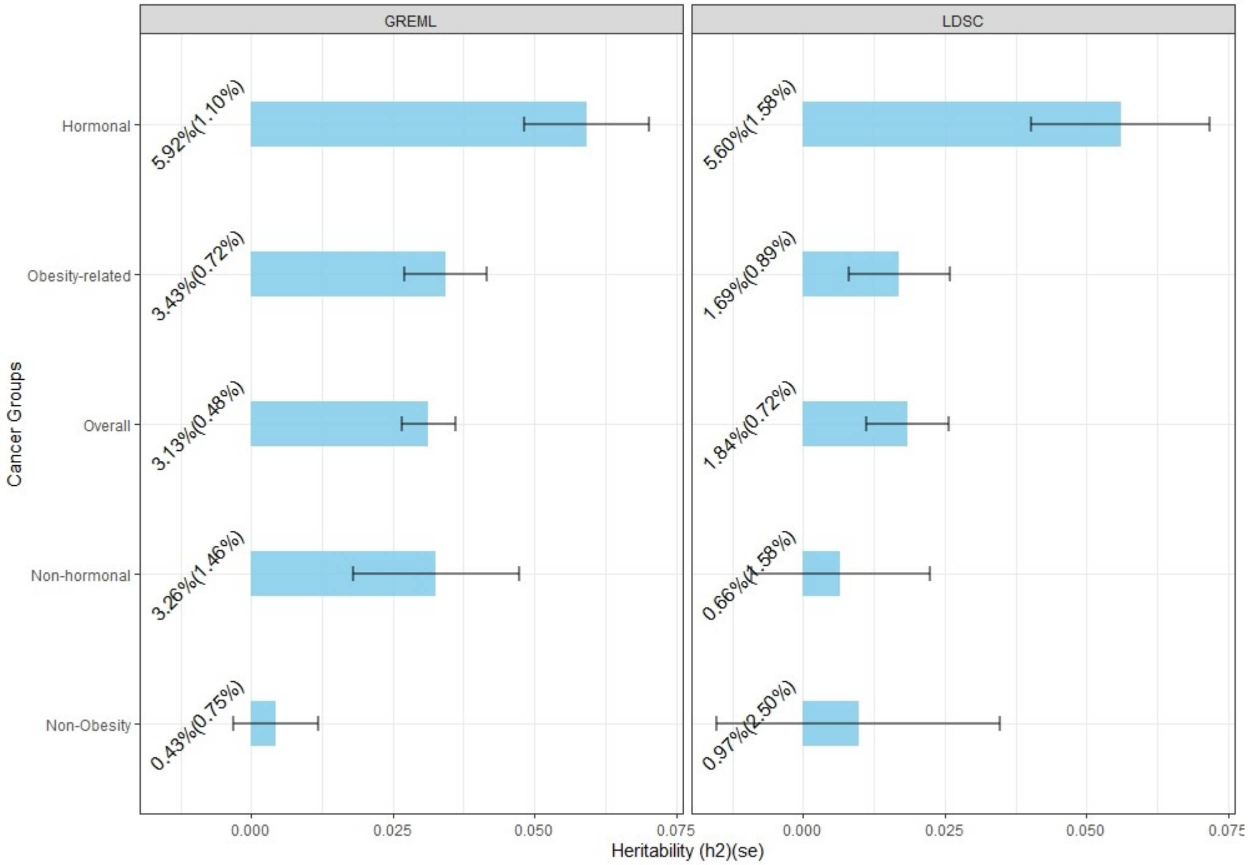

Supplementary Fig. 1: Heritability estimates for incident hormone-sensitive cancers in the UK Biobank

The panel in the left is the heritability estimate using GREML for subgroups of incident cancers indicating that the h^2 for newly diagnosed hormone-sensitive cancers is higher as compared to the rest of the cancer subgroupings. The panel in the right side is cancer subgrouping heritability estimates using summary statistics in LDSC method. The two panels consistently showed increased h^2 estimates for newly diagnosed [incident] hormone sensitive cancers. The heritability estimates with standard errors are shown in the display. The error bars are the 95% CI of the estimates.

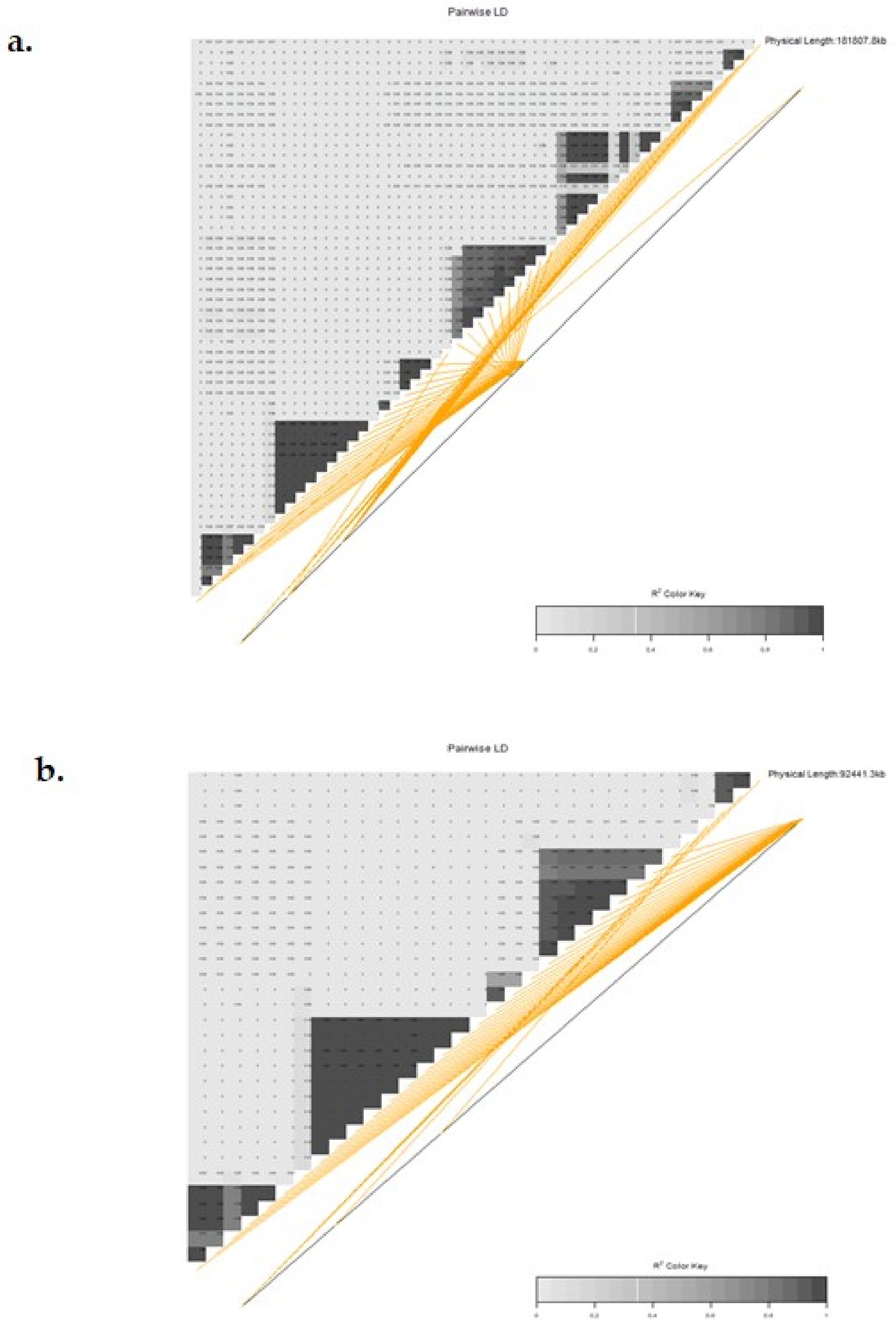

Supplementary Fig. 2: LD heatmap for pairwise LD between genome-wide significant SNPs.
Panel A [the above panel] shows the LD heatmap for pairwise LD between 55 genome-wide significant for all cases of hormone-sensitive cancer- associated SNPs. Panel B [the lower panel] is the LD heatmap between 33 genome-wide significant for incident hormone-sensitive cancer cases in the UKB.

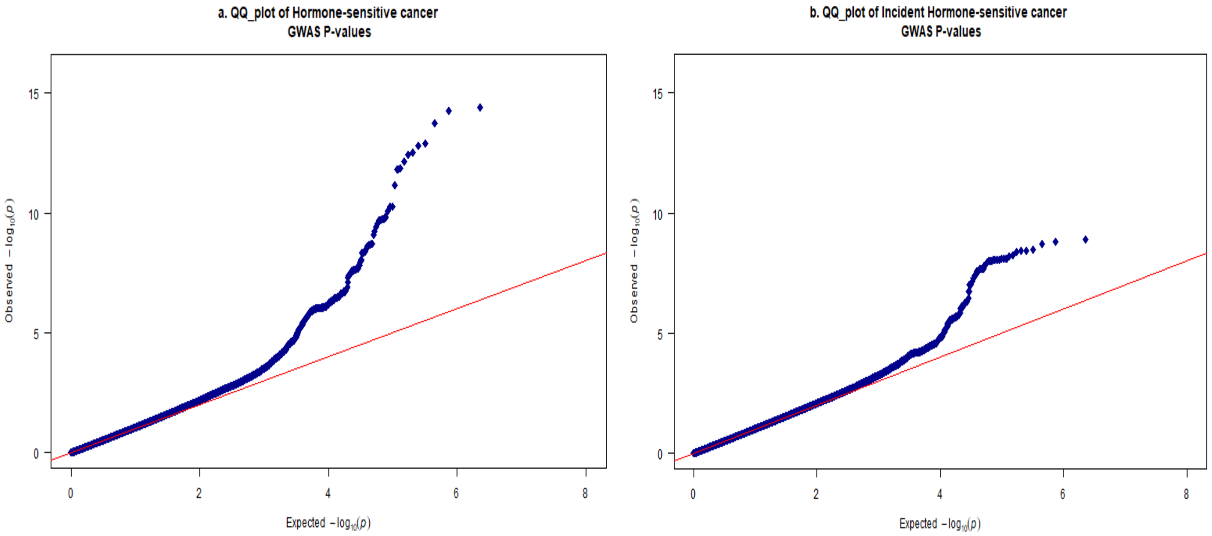

Supplementary Fig. 3: Quantile-Quantile plot of hormone-sensitive cancers association result in the UKB.

All the SNPs passed stringent quality control and all the study participants are of white British ancestry as verified by the genetic data. Panel A [the left panel] shows the overall distribution of p-values in all hormonal cancer cases. Panel B [panel in the right side] is for incident hormone-sensitive cancer cases in the UKB. In both cases the bulk of distribution is in lower part of the graph showing the absence of inflation/deflation in the genomic inflation factor (λ).

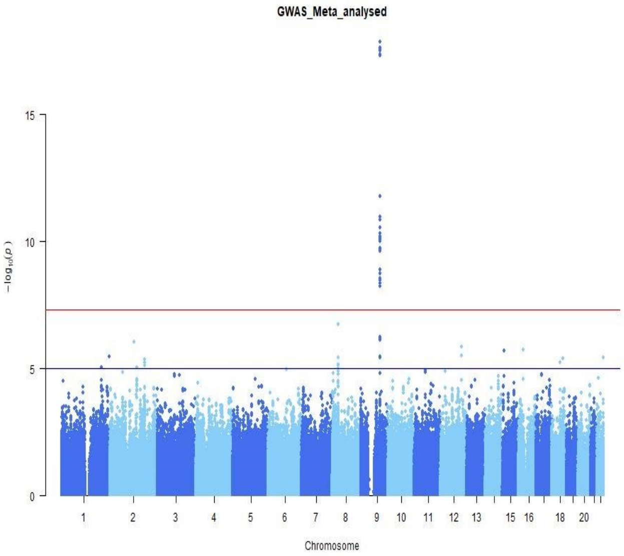

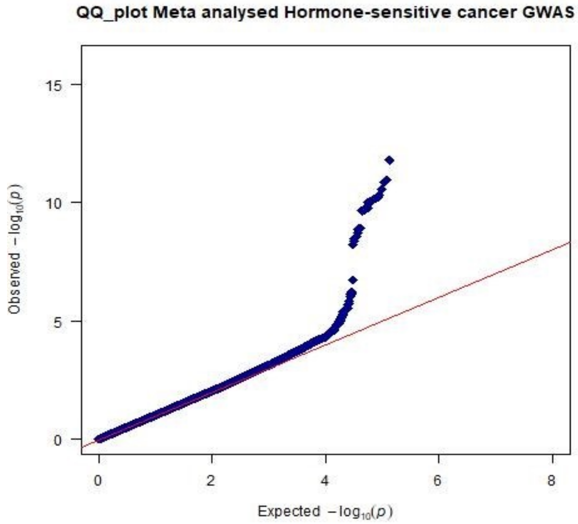

Supplementary Fig. 4: Manhattan [left panel] and Quantile-Quantile plot [right panel] for meta-analysed single trait GWAS in the UKB.

The plot in the left panel shows the Manhattan plot showing significance of each variant's association with a phenotype (components of hormone-sensitive cancer namely postmenopausal breast cancer, prostate cancer, uterine, ovarian, and thyroid cancer). The Y-axis is the negative log-base-10 of the *P* value for each of the SNPs positioned along the X-axis in genomic order by chromosomal position. The red-line shows the threshold for genome-wide significance ($P < 5x10^{-8}$), while the blue line indicates the threshold for genetic variants that showed a suggestive significance association ($P \leq 1x10^{-6}$). SNPs with the lowest *P* value of significance (i.e., highest association with meta-analysed components of hormone-sensitive cancer) are positioned at the top of the graph in the 9th chromosome. The panel in the left side shows quantile-quantile plot. This plot shows the distribution of expected P values under a null model of no significance versus observed P values. Expected -log-base-10 transformed P values (X-axis) for each association are plotted against observed values (Y-axis) to visualize the enrichment of association signal. The bulk of distribution is in lower part of the graph showing the absence of inflation/deflation in the genomic inflation factor (λ).

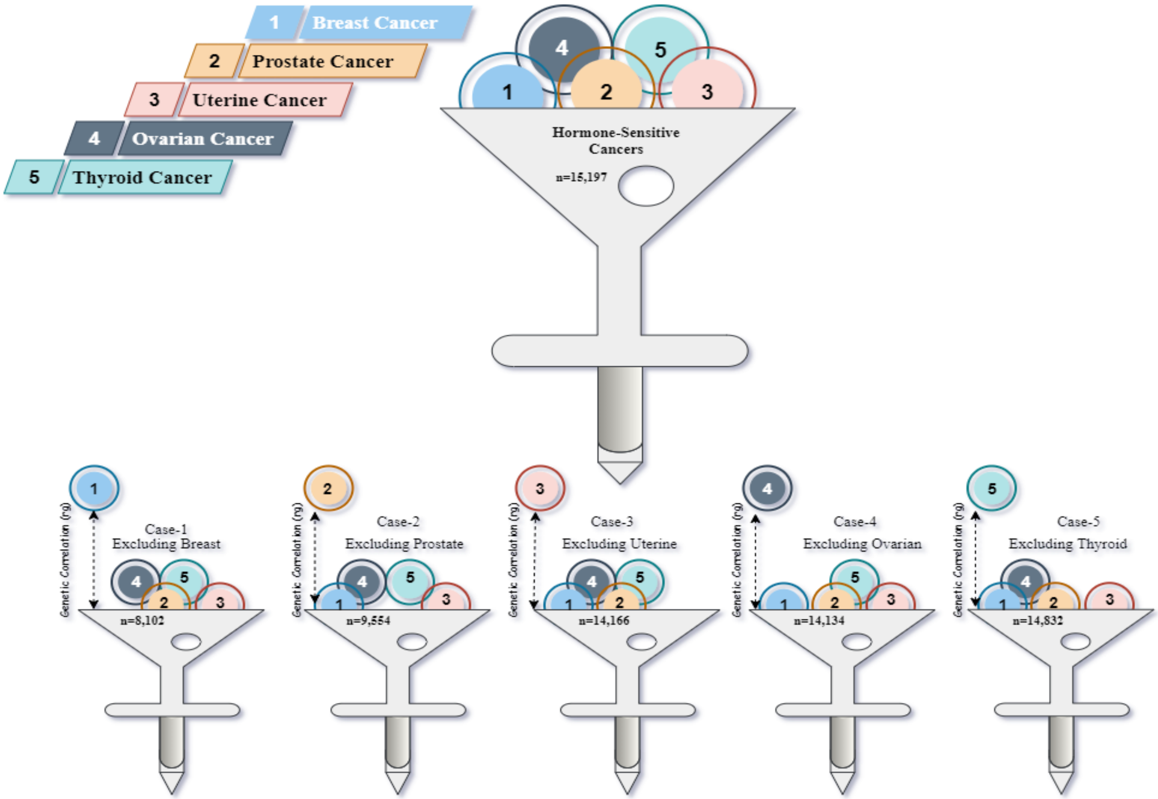

Supplementary Fig. 5: Schematic diagram of leave-one-out analysis for genetic correlation among hormone-sensitive cancers in the UK Biobank

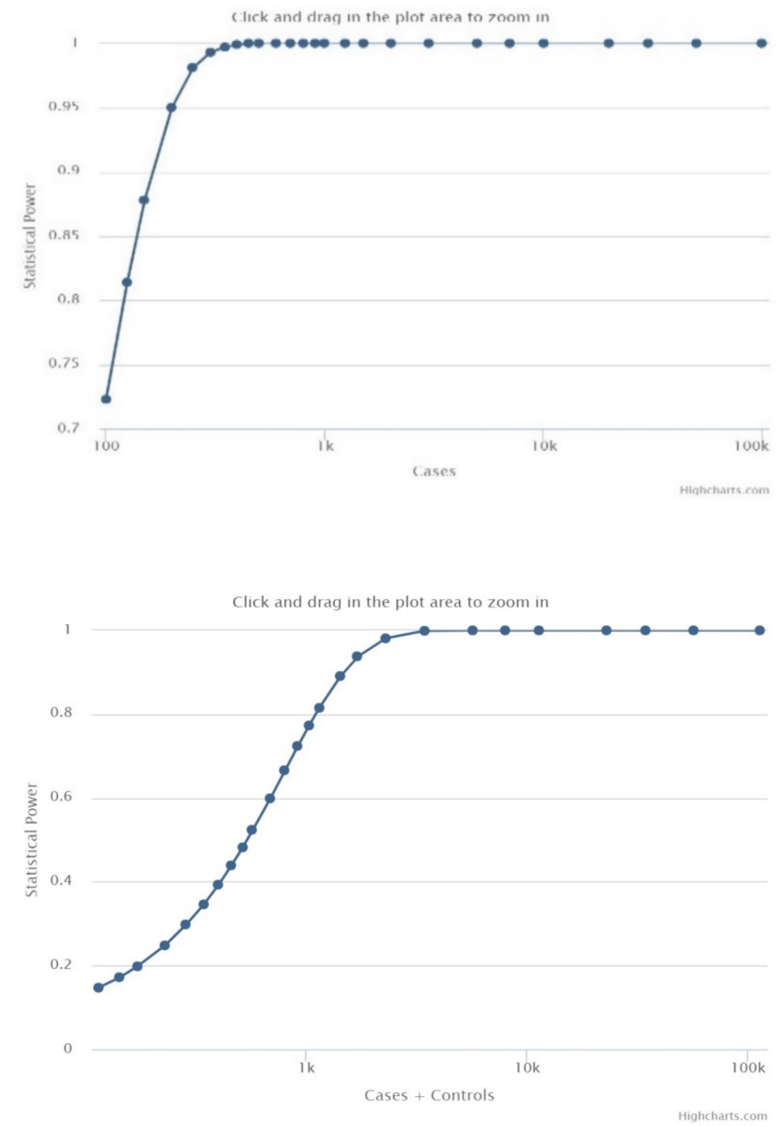

Supplementary Fig. 6: Power graph for GWAS analysis

Before the GWAS analysis, we estimate the power of the study using the genetic association study (GAS) power calculator. We applied the assumptions of number of cases and controls for hormone-sensitive cancer; the required significance level, prevalence of hormone-sensitive cancer in the sample and the disease model as additive.

Supplementary References

1. Visscher PM, Hill WG, Wray NR. Heritability in the genomics era--concepts and misconceptions. Nat Rev Genet. 2008;9(4):255-66.
2. Lee SH, van der Werf JH. MTG2: an efficient algorithm for multivariate linear mixed model analysis based on genomic information. Bioinformatics. 2016;32(9):1420-2.
3. Bulik-Sullivan B, Finucane HK, Anttila V, Gusev A, Day FR, Loh PR, et al. An atlas of genetic correlations across human diseases and traits. Nat Genet. 2015;47(11):1236-41.
4. Yang JA, Lee SH, Goddard ME, Visscher PM. GCTA: A Tool for Genome-wide Complex Trait Analysis. American Journal of Human Genetics. 2011;88(1):76-82.
