# Supplementary material for: Considering hormone-sensitive cancers as a single disease in the UK biobank reveals shared aetiology
Source: Commun Biol. 2022 Jun 21;5:614. doi: 10.1038/s42003-022-03554-y (PMC9213416; doi:10.1038/s42003-022-03554-y)
Supplement: Supplementary file 3 — Description of Additional Supplementary Files [file 42003_2022_3554_MOESM3_ESM.pdf]

Description of Additional Supplementary Files

File name: Supplementary Data 1
Description: The source data underlying the figures in the manuscript.
