# Supplementary material for: Considering hormone-sensitive cancers as a single disease in the UK biobank reveals shared aetiology
Source: Commun Biol. 2022 Jun 21;5:614. doi: 10.1038/s42003-022-03554-y (PMC9213416; doi:10.1038/s42003-022-03554-y)
Supplement: Supplementary file 5 — Reporting Summary [file 42003_2022_3554_MOESM5_ESM.pdf]

nature portfolio

Corresponding author(s): Muktar Ahmed, Sang Hong Lee

Last updated by author(s): Apr 19, 2022

Reporting Summary

Nature Portfolio wishes to improve the reproducibility of the work that we publish. This form provides structure for consistency and transparency in reporting. For further information on Nature Portfolio policies, see our Editorial Policies and the Editorial Policy Checklist.

Statistics

For all statistical analyses, confirm that the following items are present in the figure legend, table legend, main text, or Methods section.

n/a	Confirmed	
☐	☒	The exact sample size (*n*) for each experimental group/condition, given as a discrete number and unit of measurement
☐	☒	A statement on whether measurements were taken from distinct samples or whether the same sample was measured repeatedly
☐	☒	The statistical test(s) used AND whether they are one- or two-sided<br>*Only common tests should be described solely by name; describe more complex techniques in the Methods section.*
☐	☒	A description of all covariates tested
☐	☒	A description of any assumptions or corrections, such as tests of normality and adjustment for multiple comparisons
☐	☒	A full description of the statistical parameters including central tendency (e.g. means) or other basic estimates (e.g. regression coefficient) AND variation (e.g. standard deviation) or associated estimates of uncertainty (e.g. confidence intervals)
☐	☒	For null hypothesis testing, the test statistic (e.g. *F*, *t*, *r*) with confidence intervals, effect sizes, degrees of freedom and *P* value noted<br>*Give P values as exact values whenever suitable.*
☒	☐	For Bayesian analysis, information on the choice of priors and Markov chain Monte Carlo settings
☐	☒	For hierarchical and complex designs, identification of the appropriate level for tests and full reporting of outcomes
☐	☒	Estimates of effect sizes (e.g. Cohen's *d*, Pearson's *r*), indicating how they were calculated

Our web collection on statistics for biologists contains articles on many of the points above.

Software and code

Policy information about availability of computer code

Data collection	This study has been conducted using the UK Biobank Resource (http://www.ukbiobank.ac.uk) with the reference number 14575 & 20175 approved by UK Biobank.
Data analysis	Data analyses were performed using publicly available software. The links and references are provided in the manuscript. In short, we have used the well-established MTG [V2.18]software to conduct the bivariate GREML analyses and estimate the genetic correlation coefficient between each non-cancer trait and subgroups of cancer. For MTG2, the source code, executive binary file, user manual, and toy examples for practice are readily available for downloads using the link https://sites.google.com/sit/honglee0707/mtg2. The rest statistical analyses were performed using publicly available software that includes plink1.9, LDSC, and analysis packages in R & Python.

For manuscripts utilizing custom algorithms or software that are central to the research but not yet described in published literature, software must be made available to editors and reviewers. We strongly encourage code deposition in a community repository (e.g. GitHub). See the Nature Portfolio guidelines for submitting code & software for further information.

Data

Policy information about availability of data

All manuscripts must include a data availability statement. This statement should provide the following information, where applicable:

- Accession codes, unique identifiers, or web links for publicly available datasets
- A description of any restrictions on data availability
- For clinical datasets or third party data, please ensure that the statement adheres to our policy

All data will be available to approved users of the UK Biobank upon application. The data can be accessed through procedures described in the UK Biobank webpage

(http://www.ukbiobank.ac.uk/using-the-resource/). The source data underlying Figures 1-5 , Table 1-5 and supplementary figures are provided in the source data file.

Field-specific reporting

Please select the one below that is the best fit for your research. If you are not sure, read the appropriate sections before making your selection.

☒ Life sciences ☐ Behavioural & social sciences ☐ Ecological, evolutionary & environmental sciences

For a reference copy of the document with all sections, see nature.com/documents/nr-reporting-summary-flat.pdf

Life sciences study design

All studies must disclose on these points even when the disclosure is negative.

Sample size	The sample size of the study was based on the number of samples that passed genotype quality control criteria and had additional phenotype data available. Accordingly, our analyses included 235,512 controls and 15,197 hormone-sensitive cancer cases. How cancer cases were ascertained, including overall, obesity-related and hormonal cancer as prevalent and incident cases are presented in method section and Supplementary Table 6. Incident cancer cases were defined as those diagnosed after the baseline assessment before the end of follow-up (October 2016) and prevalent cases were those diagnosed before baseline assessment in the UKB.
Data exclusions	•We applied a well-established quality control procedure to exclude unreliable genotype and incomplete phenotype data. The data exclusion was set before fitting any of our models. The rationale behind data exclusion is to avoid bias in our estimates as a result of poor-quality data. The applied quality control procedure for genotype data are detailed below:<br>• To control for artifacts introduced to the data during genotyping, initial standard quality control (QC) measures were applied to all data sets before analyses. The genotype data in the UKB includes 92,693,895 SNPs genotyped from 488,377 study participants. The QC procedure for the genotypic data focused on two levels i.e. at individual and SNP level. First, at the individual level, we exclude individuals with a call rate of less than 95% and individuals who did not self-identify as white British ancestry or who exhibited sex inconsistencies (sex mismatch between self-reported phenotype sex and genotype determined sex data) and had a putative sex chromosome aneuploidy (chromosomal anomalies). To check identical genes shared through common ancestors, we randomly selected individuals from a pair and excluded those pairs in which their genomic relationship is larger than 0.05. Furthermore, to avoid bias induced as a result of population stratification and to ensure participants are taken from a relatively homogenous population, we checked the population substructure in the Principal Component (PC) analysis to the excluded individual as population outliers with the first or second PC outside ± 6 SD of the population mean. Based on the release of the UKB genotype dataset, for those who were included in both the first and second, we calculated the genotype discordance rate between imputed genotype of the two versions for each SNP and each individual and exclude those with a genotype discordance rate of more than 0.05. Secondly at the SNP level, genetic markers with an INFO score <0.6, markers that deviate significantly from Hardy-Weinberg equilibrium (HWE) (1.00E-07) or with a call rate <0.95, with MAF <0.01 and ambiguous or duplicated SNPs were excluded. To avoid systematic differences between cases and controls being interpreted as genetic variance, a more stringent quality-control process was then applied to the data. This included excluding individuals with incomplete phenotype data and re-moving markers with a minor allele frequency of less than 1%. In this study, we used high-quality SNPs from the International HapMap Project [HapMap3] that were reliable in estimating genetic variance and covariance at the genome-wide level, feasible for more complicated analyses and there was no substantial difference between estimated genetic variance from HapMap3 and 1000 genome SNPs. After QC, 1,217,312 HapMap3 SNPs with 288,837 study participants have remained for the analyses.
Replication	We divided the UK Biobank working dataset into three subsets and perform the analysis, then validate the three estimates for each type of analyses, implying these findings are replicable.
Randomization	We randomly split the UK Biobank working dataset for replication and meta-analysed the estimate across the three datasets.
Blinding	Blinding was not applicable to our study, because the data were collected by the UK Biobank and we used the deidentified individual level information. In addition, genetic association testing does not require blinding.

Reporting for specific materials, systems and methods

We require information from authors about some types of materials, experimental systems and methods used in many studies. Here, indicate whether each material, system or method listed is relevant to your study. If you are not sure if a list item applies to your research, read the appropriate section before selecting a response.

Materials & experimental systems

n/a	Involved in the study	
☒	☐	Antibodies
☒	☐	Eukaryotic cell lines
☒	☐	Palaeontology and archaeology
☒	☐	Animals and other organisms
☐	☒	Human research participants
☒	☐	Clinical data
☒	☐	Dual use research of concern

Methods

n/a	Involved in the study	
☒	☐	ChIP-seq
☒	☐	Flow cytometry
☒	☐	MRI-based neuroimaging

Human research participants

Policy information about studies involving human research participants

Population characteristics	We used data in the UK Biobank (UKB) (http://www.ukbiobank.ac.uk). All experiments in this study were conducted in accordance with respective ethical guidelines and regulations. The UKB is a large prospective study that aims to improve the diagnosis, treatment, and prevention of disease. It holds detailed demographic, social and medical information for more than 500,000 participants age 37-73 years at recruitment conducted between 2006-2010, along with physical measures, such as weight and blood pressure. Participants' consent was obtained during enrolment, with permission for access to their medical and other health-related data for research purposes.
Recruitment	Between 2006 and 2010, the UK Biobank collected detailed data on 502,650 people. They used 22 assessment centres based in England, Scotland and Wales. Participants were recruited from National Health Service (NHS) patient registers and contacted if they lived within a reasonable proximity to an assessment centre. The age group involves people at risk over the next few decades of developing a wide range of important diseases, conditions and covariates. The phenotype and genotype information are based on extensive baseline questionnaire and physical measures, as well as stored blood and urine sample that allow many different types of assay, incorporated with information from the UK National Health service.
Ethics oversight	The UK Biobank's scientific protocol has been reviewed and approved by the North West Multi-Centre Research Ethics Committee (MREC), which covers the UK. It also sought the approval in England and Wales from the Patient Information Advisory Group (PIAG) for gaining access to information that would allow it to invite people to participate. PIAG has since been replaced by the National Information Governance Board for Health & Social Care (NIGB). In Scotland, UK Biobank has approval from the Community Health Index Advisory Group (CHIAG).

Note that full information on the approval of the study protocol must also be provided in the manuscript.
